# An online identification approach for ship domain model based on AIS data

**DOI:** 10.1371/journal.pone.0265266

**Published:** 2022-03-10

**Authors:** Wei Zhou, Jian Zheng, Yingjie Xiao

**Affiliations:** 1 Merchant Marine College, Shanghai Maritime University, Shanghai, China; 2 Engineering Research Center of Simulation Technology, Ministry of Education, Shanghai, China; 3 College of Transport and Communications, Shanghai Maritime University, Shanghai, China; Tongji University, CHINA

## Abstract

As an important basis of navigation safety decisions, ship domains have always been a pilot concern. In the past, model parameters were usually obtained from statistics of massive historical cumulative data, but the results were mostly historical analysis and static data, which obviously could not meet the needs of pilots who wish to master the ship domain in real time. To obtain and update the ship domain parameter online in time and meet the real-time needs of maritime applications, this paper obtains CRI as the weight coefficient-based PSO-LSSVM method and proposes to use short-term AIS data accumulation through the risk-weighted least squares method online rolling identification method, which can filter nonhazardous targets and improve the identification accuracy and real-time performance of nonlinear models in the ship domain. The experimental examples show that the method can generate the ship domain dynamically in real time. At the same time, the method can be used to study the dynamic evolution characteristics of the ship domain over the course of navigation, which provides a reference for navigation safety decisions and the analysis of ship navigation behavior.

## 1. Introduction

The ship domain is an important concept of water transportation, which was defined in 1975 by Goodwin [1]. To ensure their own safety, ships need to maintain a certain safe distance from surrounding ships during navigation, and the ship domain model is the way to describe this space scope [2]. Research on ship domain models has experienced decades of development. Building a practical ship domain model can better describe ship behavior. For example, Reference [3] proposes the novel concept of the probabilistic ship domain, which depicts the ship domain boundary as a vague value. An adaptive ship safety domain is proposed with spatial risk functions in Reference [4]. The reasonableness and superiority of establishing a ship domain model considering the factors affecting both one’s own ship and other ships are analyzed in Reference [5]. Reference [6] presents a new ship domain model that is both realistic and practical, including various factors considered by seafarers based on the awareness values formed. In Reference [7], a free-form ship domain was developed empirically for navigation in confined waters, and the size of the ship domains was assumed to be dynamically enlarged with increased ship speeds. Through the use of a machine learning algorithm, Reference [8] develops intelligent ship domain models, which better represent the usual navigation practice than traditional approaches. From the perspective of the development process of ship models, their research methods and shapes have gone through a process from experience, statistics, analytical expression, data mining and intelligent technology, from simple geometric shapes to complex shapes, and from static to dynamic shapes [9].

The utilization of AIS information has received increasing attention, and many scholars have begun to use massive AIS data to analyze and obtain ship domain models [10,11]. Reference [12] observes ships sailing during a four-year period by AIS, estimates how closely ships pass each other and fixed objects, and then establishes an empirical minimum ship domain. Considering the navigation characteristics of ships with limited maneuvering capability and the influence of ships on the ship effect, an algorithm to determine the boundary of the ship domain model is proposed, and experiments are carried out using AIS trajectory data in Reference [13]. According to Reference [14], the surrounding waters of the target ship are divided into grids, and then the grid densities of ships are calculated to determine the shape and size of the ship domain. By analyzing a large number of ship-encounter samples obtained from AIS data, the available maneuvering margin (AMM) can be used in Reference [15] to explain the first evasive maneuvere, and finally, the size of the ship domain can be empirically estimated. From Reference [16], through AIS data, the relationship between ship-avoidance behavior and the nearest encounter point is analyzed; the ship-avoidance behavior is quantified, and then the ship domain boundary is obtained. In Reference [17], the method was proposed by using a large volume of AIS data to obtain the ship domains in restricted waters.

The above research results provide ideas and references for obtaining ship domain models using AIS data, but most of them are statistical analyses of diachronic data, lacking real-time dynamic research. With the need for real-time information navigation applications, it is necessary to propose a more dynamic and real-time ship domain model acquisition method. Different from traditional statistical methods, the method in this paper makes AIS data serve navigation safety better, enables ships to quickly perceive the dynamics of surrounding ships, identifies the actual parameters of the ship domain, and generates the boundary of the ship domain so that pilots can grasp the dynamics of their own ship domain in time and provide a basis for their navigation safety decisions more quickly. The main work of this paper is as follows:

The idea of generating ship domains online is proposed to provide a reference for the dynamic application of AIS data in navigation safety decision making. Short-term accumulated AIS data are adopted. When the minimum requirements for identification data are met, the collision risk index, which is obtained by the PSO-LSSVM method, is used as the control method of identification error filtering, combined with the weighted least square method, to quickly generate the ship domain and carry out dynamic rolling updates. In this paper, starting from a period of the navigation process, the change in the ship domain brought by the change in ship speed and navigation water area in the dynamic navigation process is observed to discover the evolution law of the ship domain, grasp its dynamic change in time, and provide a reference for an in-depth and detailed study of its change.

The rest of this paper is organized as follows: Section 2 introduces the ship domain model to be identified, considers the data required for model identification using AIS, and describes the corresponding ship-encounter parameter calculation formula. Then, Section 3 proposes a schematic diagram of online identification, including single online identification and real-time online rolling identification. In Section 4, the PSO-LSSVM method for collision risk index estimation is introduced, and the identification method of collision risk weighting is established. Then, in Section 5, an experimental example combined with the application of this method is described, and the results are analysed. In Section 6, based on the experimental results, some dynamic evolution laws of the ship domain in navigation are summarized. Finally, Section 7 summarizes the main findings and applications of the methods described in this paper, as well as suggestions for future work.

## 2. Ship domain model and parameter calculation

### 2.1. Basic model of the ship domain

In terms of ship domain shapes, ship domain models usually include circular, fan-shaped, elliptical, quasi-elliptical, polygonal, etc. Davis [18] proposed the circular ship domain, and Goodwin proposed a similar fan-shaped ship domain [19]. The Fujii model [20], Goldwell model [21], Kijima model [22], and quaternion ship domain model [23] are elliptic or quasi-elliptic ship domain models, while the polygonal ship domain is proposed in References [24,25]. To meet the need for fast online identification, the mainstream elliptic model is used as the basis for ship domain model identification. The elliptic equation is as follows:

$$\frac{{\left(x-x_{0}\right)}^{2}}{a^{2}}+\frac{{\left(y-y_{0}\right)}^{2}}{b^{2}}=1$$ (1)

where a, b and (*x*_0_, *y*_0_) are the main parameters to be identified. According to the equation-solving requirements, at least four or more target ship data are required to identify model parameters. To meet the identification accuracy requirement, the position of the target ship should be distributed in four quadrants of the coordinate system around the ship, not concentrated in one or a few quadrants. Considering that the identification model is nonlinear, to improve the accuracy and efficiency of identification, this paper adopts the collision risk weighted least square method to carry out online identification.

### 2.2. Ship encounter situation parameters

AIS data contain dynamic information required by ship-encounter situations, which is the basis for identifying ship domain models. Only by calculating the ship encounter parameters can the superposition of the target ships around the ship be dynamically grasped, the situation distribution of the ship encounter be obtained, the required data for solving the domain model equation be obtained, and then the model parameters can be identified. The parameters are calculated as follows [26]:

Suppose that the corresponding coordinates of the longitude and latitude of the ship are (*x*_O_, *y*_O_); the speed is *v*_O_; the course is *C*_O_, and the corresponding coordinates of the longitude and latitude of the target ship are (*x*_T_, *y*_T_); its speed is *v*_T_, and its course is *C*_T_.

$$D=\sqrt{{\left(x_{T}-x_{O}\right)}^{2}+{\left(y_{T}-y_{O}\right)}^{2}}$$ (2)

$$Q=arctan\left(\frac{x_{T}-x_{O}}{y_{T}-y_{O}}\right)$$ (3)

$$K=\frac{V_{T}}{V_{O}}$$ (4)

$$V_{r}=\sqrt{V_{O}^{2}+V_{T}^{2}-2{cos(C_{O}-C_{T})V_{O}V}_{T}}$$ (5)

$$C_{r}=arccos\left(\frac{V_{O}-V_{T}cos(C_{O}{-C}_{T})}{V_{r}}\right)$$ (6)

$$DCPA=D∙sin(C_{r}-q-\pi )$$ (7)

$$TCPA=\frac{D∙cos(C_{r}-q-\pi )}{V_{r}}$$ (8)

Considering the requirement of position superposition processing of the target ship, the position distribution of the target ship around the ship is marked by distance using Eq (2) and relative bearing using Eq (3). In Eq (3), the value of the arctangent function in the calculation formula of the relative bearing should be determined according to the coordinate quadrant where the target ship is relative to the center of the ship, and the angle value is [0,360°]. K is the ratio of ship speed using Eq (4). *V*_*r*_ is the relative speed using Eq (5). *C*_*r*_ is relative course using Eq (6). DCPA and TCPA can also be obtained using Eqs (7) and (8). In addition, the main parameters of this paper are shown in Table 1.

**Table 1:** The main parameters in this paper.

Parameters	Parameters Mean	Unit or Range
*a*	Major axis of the ship domain	*nautical mile*
b	Minor axis of the ship domain	*nautical mile*
(*x*_0_, *y*_0_)	Central coordinates of the ship domain	*nautical mile*
D	Relative distance between own ship and target ship	*nautical mile*
*Q*	Relative bearing of the target ship relative to own ship	degree
*K*	Speed ratio of the target ship to own ship	ratio
DCPA	Minimum encounter distance	*nautical mile*
*TCPA*	Minimum encounter time	*hour*
CRI	Risk Index of collision between target vessel and own vessel	[0,1]

## 3. Schematic

As the parameters are identified online and need to be updated dynamically, only a small amount of data can be relied on to obtain the final results. This is significantly different from the results obtained from massive data mining. Therefore, it is necessary to solve the problem of reliability of the method and meet the requirement of the minimum dataset required for identification. Based on the above considerations, this paper proposes the following identification schematic.

The core idea is to achieve the required dataset identification through short-term data accumulation. The dataset is updated by rolling to meet the requirement of real-time results. Differential data processing can be realized through collision risk to improve the identification progress. At the same time, the average collision risk and the number of identification data points were used as references for the reliability of the conclusions. Combined with the average collision risk and dynamic identification results, the pilot can better grasp the real-time dynamics of the ship domain. Compared with the traditional model, the pilot can also understand the safety margin of the compressible space in the ship domain. The results are a research method of the dynamic evolution of the ship domain and a reference for navigation safety decision-making. The schematic diagram includes single identification and dynamic rolling identification.

### 3.1. Online single identification

1. Through the VTS center of the maritime administration of the jurisdiction, AIS data can be obtained. Then, according to the AIS data, the meeting situation between the target ship in the surrounding waters and the ship can be obtained through dynamic and real-time calculations. Considering the general range of the ship domain, the boundary is 3 nautical miles for open waters and 1.5 nautical miles for narrow waters.
2. DCPA, TCPA, relative distance D, relative bearing Q, velocity ratio K and other parameters are calculated dynamically. The ship collision risk CRI is obtained by the PSO-LSSVM method, and the risk of the target ship is graded.
3. With a unit time interval, ships that meet the requirements of the encounter range are identified as target ships and alternatives that can participate in the identification data of the ship domain model.
4. When the number of identified target ships reaches the required number of ships for model identification, online parameter identification can be carried out quickly; the current ship domain model parameters can be dynamically updated, and the ship domain boundary curve can be drawn.
5. To avoid the identification error caused by the small amount of data, ship collision risk is adopted as the method of filtering nonhazardous targets. As the error weight coefficient of identification, CRI can control the influence of nonhazardous targets on the results so that the dangerous targets are reflected in the identification results, and the meaning of the ship domain as the scope of ship navigation safety is better reflected.

**Fig 1:**
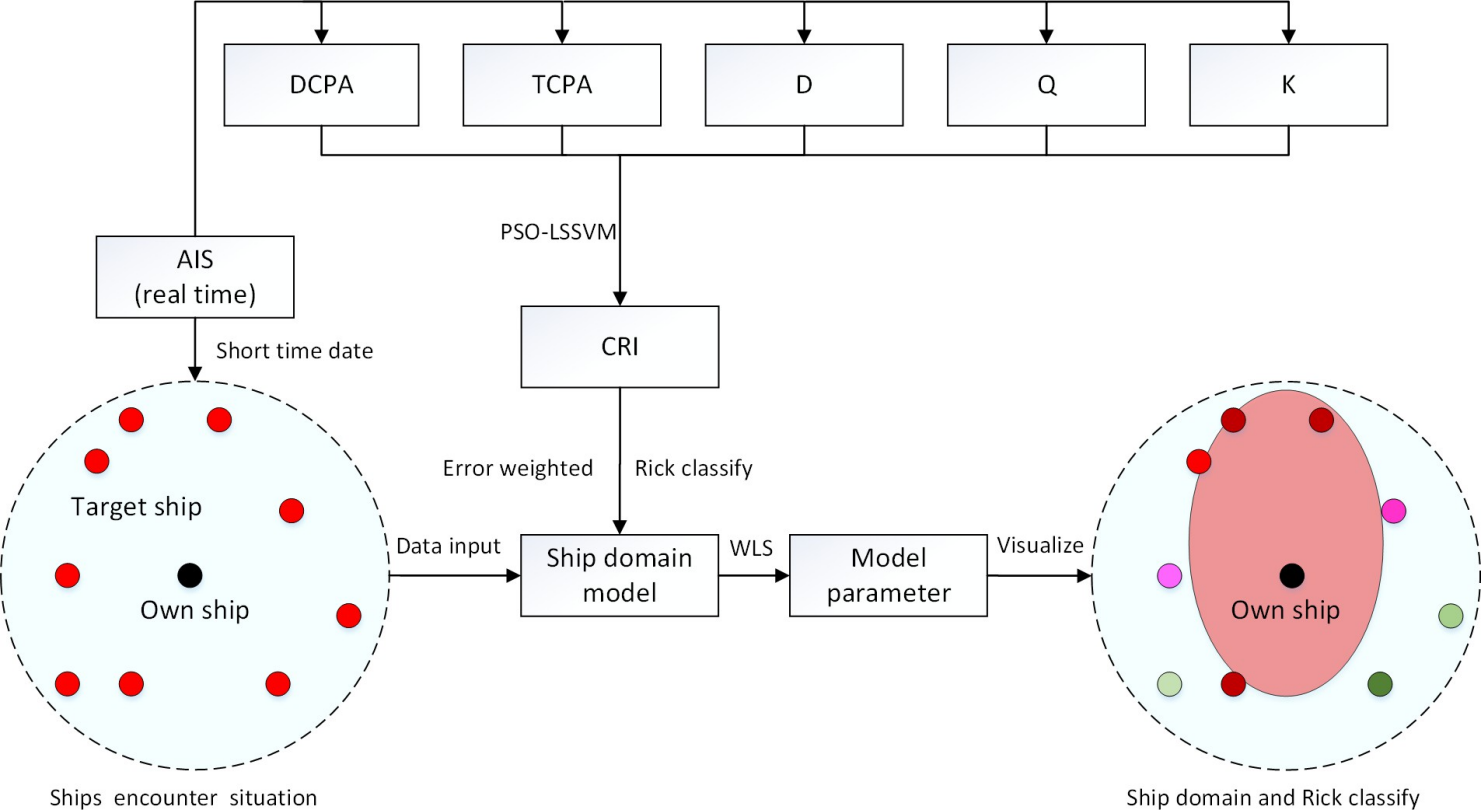
Schematic diagram of online identification of the ship domain.

According to the above process, when the ship domain elliptic equation can be identified and the error accuracy requirements are met, the ship domain identification results can be dynamically output. The identification schematic diagram is shown in Fig 1.

### 3.2. Real-time online rolling identification

**Fig 2:**
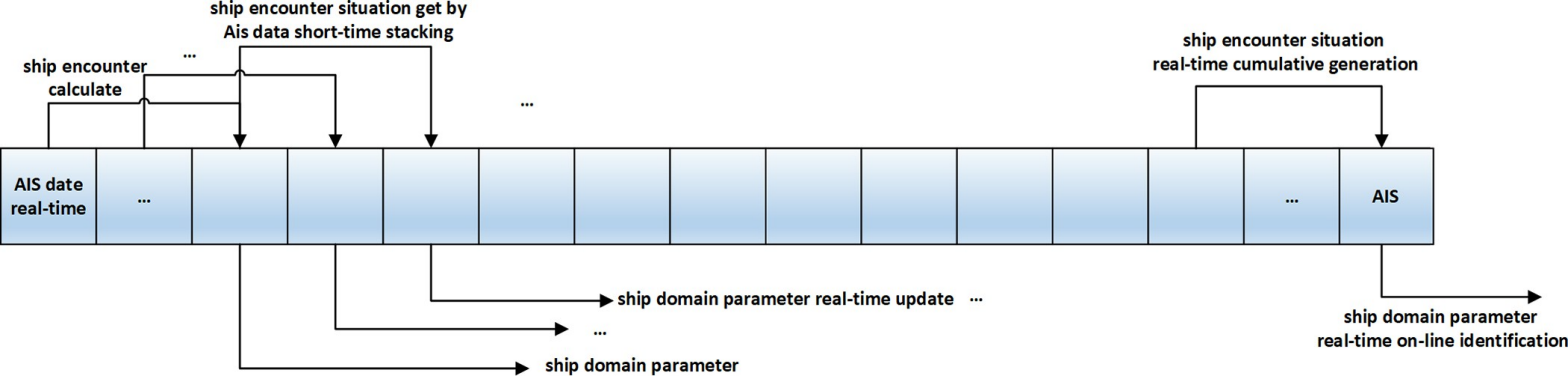
Schematic diagram of real-time online rolling identification based on AIS data.

1. AIS data are received in real time, and when the accumulated data requirements are met, the encounter situation of ships is superimposed, and the ship domain is identified according to Fig 1.
2. According to the schematic method in Fig 2, real-time rolling identification of the ship domain can be realized by iterating real-time data and replacing earlier duration data to dynamically update the ship domain model for the pilot.

## 4. Methods

### 4.1. PSO-LSSVM for collision risk index estimation

CRI, namely, the collision risk index, is a parameter used to measure the degree of ship collision risk. Usually, the value range can be [0, 1]. The higher the value, the more dangerous it is. The DCPA, TCPA, relative distance D, relative bearing Q and ship speed ratio K can be used to measure the relevant indicators of ship collision risk. The indicator value and weight of CRI are related to the judgment of expert experience, and this paper refers to the fuzzy calculation result of referring to the survey results of experts in References [27,28] as a training data sample. Then, CRI is obtained through PSO-LSSVM. This method has good small-sample generalization learning ability and can well reflect the expert experience in the numerical results of CRI.

#### 4.1.1. PSO

Particle swarm optimization (PSO) is a fast iterative algorithm to find the optimal particle in the search area and the search for the optimal particle as the solution of the optimization problem [29]. In the iterative process, the individual extremum *p*_*best*_ and global extremum *g*_*best*_ are constantly updated, and the particles are constantly updated accordingly. The *i* particle in the *n* dimension is represented as *x*_*i*_ = (*x*_*i*1_, *x*_*i*2_,⋯,*x*_*in*_), the speed is (*v*_*i*1_, *v*_*i*2_,⋯,*v*_*in*_), the corresponding individual extremum is *p*_*best*_ = (*p*_*i*1_, *p*_*i*2_,⋯,*p*_*in*_), and the global extremum of the particle swarm is *p*_*best*_ = (*p*_*i*1_, *p*_*i*2_,⋯,*p*_*in*_). The velocity and position of particles are constantly updated with the extreme value, which are calculated as Eq (9), and finally find the optimal solution.

$$\left\{ \begin{array}{c} v_{i}^{k}=\omega v_{i}^{k-1}+c_{1}r_{1}(p_{best}-x_{i}^{k-1})+c_{2}r_{2}(g_{best}-x_{i}^{k-1})\\ x_{i}^{k}=x_{i}^{k-1}+v_{i}^{k} \end{array}\right.$$ (9)

where $v_{i}^{k}$ is the velocity of particle *i* in iteration *k*; $x_{i}^{k}$ is the corresponding position; *ω* is the momentum coefficient; *c*_1_、*c*_2_ is the learning factor, *c*_1_、*c*_2_∈(0,2); and *r*_1_、*r*_2_ is a random number between (0,1).

#### 4.1.2. LSSVM

The least squares support vector machine (LSSVM) model is an improvement on the standard SVM. LSSVM uses the equality constraint and linear solution to replace the inequality constraint and nonlinear solution in SVM, which has higher accuracy and efficiency. To train sample set {(***x***_*i*_, y_i_)|*i* = 1,2,⋯,*n*}, ***x***_*i*_∈***R***^*m*^, *y*_*i*_∈***R***, ***x***_*i*_ *is* input vector, *y*_*i*_ is the output value, and *n* is the number of training samples. High-dimensional nonlinear mapping is **φ**: ***R***^*N*^→***H***, where ***H*** is a high-dimensional characteristic space, in which the sample set is as follows:

$$y_{i}=w^{T}\varphi (x_{i})+b$$ (10)

where ***w*** is the weight vector and b is the value of the offset.

According to the principle of minimizing structural risk, the objective function of the LSSVM optimization problem is established as follows [30]:

$$\underset{w,b,\xi }{\min}\frac{1}{2}{\left\|w\right\|}^{2}+\frac{1}{2}\gamma \sum_{i=1}^{n}{\xi_{i}}^{2}$$ (11)

The constraint condition is as follows:

$$y_{i}=w^{T}\varphi (x_{i})+b+\xi_{i}$$ (12)

where ***w*** is the sample weight vector; *ξ*_*i*_ is the sample relaxation variable; and *γ* is the penalty factor for error.

The Lagrange solution equation of the objective function is as follows:

$$L(w,b,\xi ,\alpha )={\frac{1}{2}\|w\|}^{2}+\frac{1}{2}\gamma \sum_{i=1}^{n}{\xi_{i}}^{2}-\sum_{i=1}^{n}\alpha_{i}[w^{T}\varphi (x_{i})+b+\xi_{i}-y_{i}]$$ (13)

where *α*_*i*_ is the Lagrange multiplier. The optimal parameters *α* and b can be obtained by the following KKT conditions. [31]

$$\left\{ \begin{array}{l} \frac{\partial L}{\partial b}=0⟹\sum_{i=1}^{n}\alpha_{i}=0\\ \frac{\partial L}{\partial \xi }=0⟹\alpha_{i}=\gamma \xi_{i}\\ \frac{\partial L}{\partial w}=0⟹w=\sum_{i=1}^{n}\alpha_{i}\varphi (x_{i})\\ \frac{\partial L}{\partial \alpha }=0⟹w^{T}\varphi (x_{i})+b+\xi_{i}-y_{i}=0 \end{array}\right.$$ (14)

When ***w*** and *ξ*_*i*_ of the characteristic space are eliminated, the optimization problem is transformed as follows:

$$\left[\begin{array}{cc} 0 & \Theta^{T}\\ \Theta & K+\gamma^{-1}I \end{array}][\begin{array}{l} b\\ \alpha \end{array}]=[\begin{array}{l} 0\\ y \end{array}\right]$$ (15)

where ***I*** is the *n* order identity matrix, **Θ** = [1,2,⋯,*n*], *α* = [*α*_1_, *α*_2_,⋯,*α*_*n*_], ***y*** = [*y*_1_, *y*_2_,⋯,*y*_*n*_], and ***K*** is the kernel function matrix.


$$K_{ij}={\varphi (x_{i})}^{T}\varphi (x_{j})$$ (16)

According to Eqs (15) and (16), the regression function of LSSVM is [32]:

$$y_{i}=\sum_{i=1}^{n}\alpha_{i}K({x,x}_{i})+b$$ (17)

**Fig 3:**
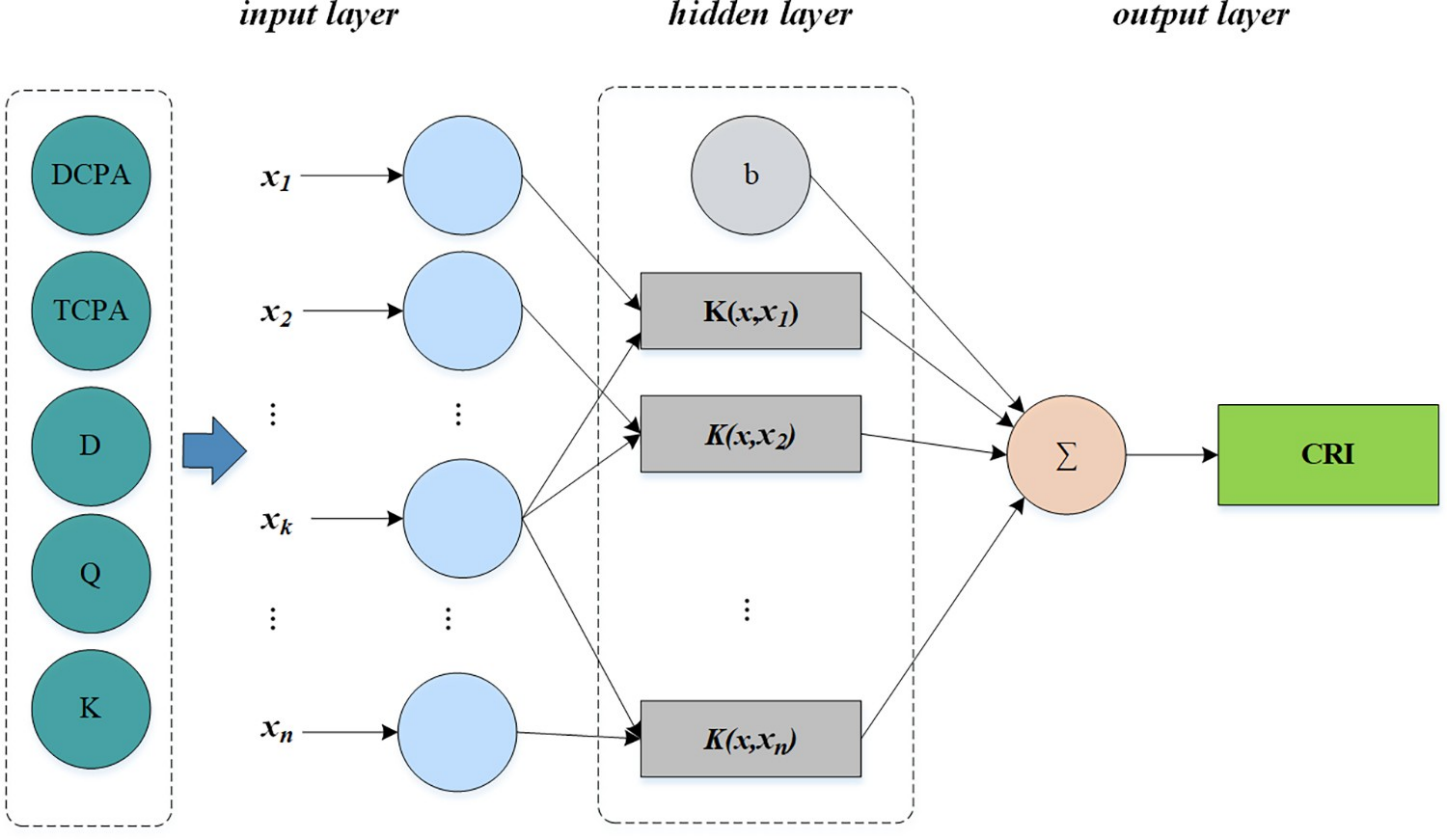
Schematic diagram of the CRI model based on the LSSVM.

If the CRI is acquired by the LSSVM, its model diagram is shown in Fig 3.

The kernel function of the LSSVM method in this paper is the Gaussian radial basis function (RBF), and the equation is as follows [33]:

$$K(x,x_{i})=exp\left(\frac{-\|x-x_{i}\|}{2\sigma^{2}}\right),\sigma >0$$ (18)

where *σ* is the kernel function parameter. The smaller the value is, the easier it is to underfit, and the larger the value is, the easier it is to overfit. Because of its simple structure and strong generalization ability, the RBF function can satisfy the rapid optimization of parameters.

#### 4.1.3. CRI acquisition by PSO-LSSVM method

**Fig 4:**
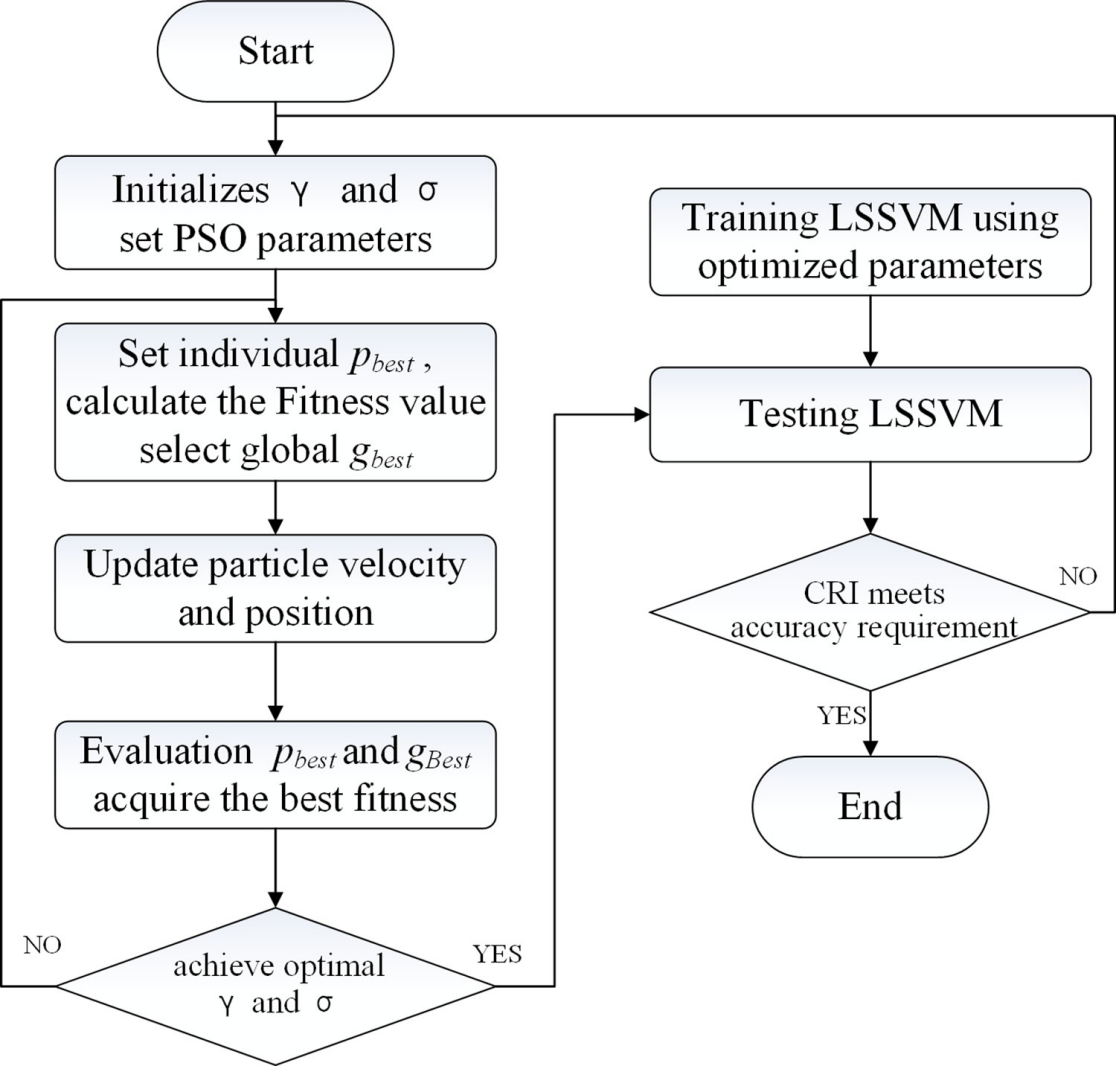
The flow of CRI acquisition by PSO-LSSVM.

The accuracy of the LSSVM method depends on the values of the kernel parameter *σ* and the penalty factor *γ*. The PSO algorithm can quickly find the optimal *σ* and *γ* and reduce the tedious parameter adjustment operation [34]. The flow of CRI acquisition by PSO-LSSVM is shown in Fig 4, and steps are as follows:

Step 1. The parameters *σ* and *γ* in the LSSVM are initialized, and the velocity and position of each particle are determined in PSO.

Step 2. The LSSVM method is used to train each particle, and the training results find the optimal position, whose value is equal to the maximum value of the fitness function. The RMSE, namely, the root mean square error, is taken as the fitness function, and the equation is as follows [35]:

$$RMSE=\sqrt{\frac{\sum_{i=1}^{N}{\left(y_{i}-\hat{y_{i}}\right)}^{2}}{N}}$$ (19)

where *y*_*i*_ and $\hat{y_{i}}$ are the real value and corresponding predicted value of the model respectively, and the training and testing datasets need to be normalized.

Step 3. The particle position and velocity are changed according to the position and velocity update algorithm.

Step 4. The objective function value of the particle is calculated: the objective function value calculated by each particle is compared with its optimal value. If it is better than the historical optimal objective function, the historical optimal value is replaced by the objective function of the current particle; otherwise, the original value is still used, and the population optimal objective function is evaluated in the same way.

Step 5. If the maximum number of iterations is reached or the error is less than the set value, the iteration is terminated; otherwise, return to Step 2.

Step 6. LSSVM is established and tested according to the optimal parameters. If the CRI error meets the requirements, the program is terminated, and the final PSO-LSSVM model is obtained; otherwise, return to Step 1.

### 4.2. Collision risk weighted identification method

**Fig 5:**
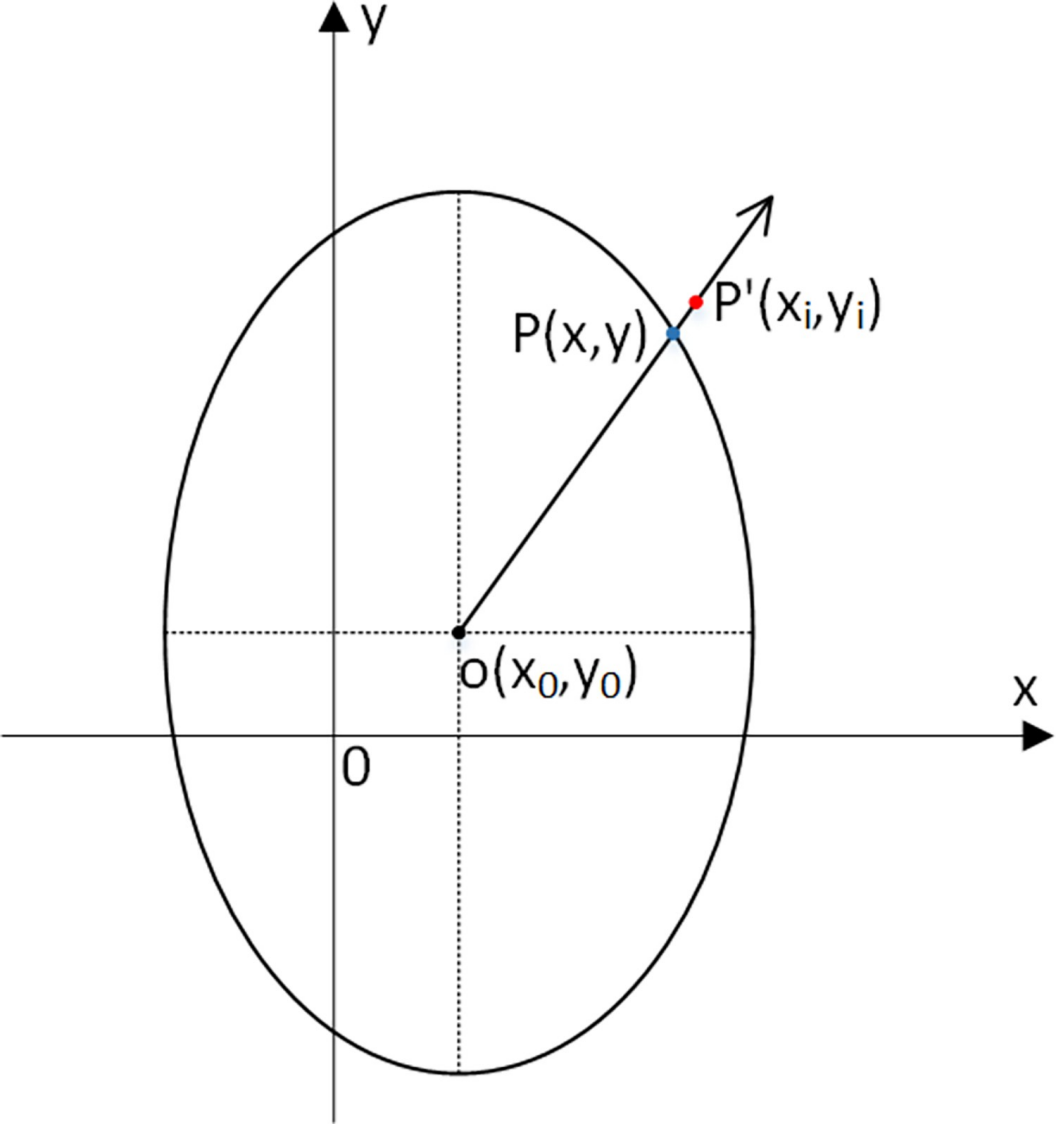
Relationship between the identified ship domain model boundary and AIS data.

Considering that the elliptical ship domain is adopted as the identification object in this paper, as shown in Fig 5, the coordinate of AIS data point P’ is (*x*_*i*_, *y*_*i*_); the coordinate of point P on the boundary of the identified ellipse domain model is (*x*, *y*), and the distances from to the coordinate (*x*_0_, *y*_0_) of the ellipse center point O are *d*_*i*_ and *d*, respectively.


$$d_{i}=\sqrt{{\left(x_{i}-x_{0}\right)}^{2}+{\left(y_{i}-y_{0}\right)}^{2}}$$ (20)

$$d=\sqrt{{\left(x-x_{0}\right)}^{2}+{\left(y-y_{0}\right)}^{2}}$$ (21)

The geometric distance between AIS data points P’ and P on the boundary of the identified elliptic ship domain model is as follows:

$$\Delta d=|\sqrt{{\left(x_{i}-x_{0}\right)}^{2}+{\left(y_{i}-y_{0}\right)}^{2}}-\sqrt{{\left(x-x_{0}\right)}^{2}+{\left(y-y_{0}\right)}^{2}}|$$ (22)

When line OP’ is perpendicular to the X axis, *x*_*i*_ = *x*_0_ and *x* = *x*_0_ are substituted into Eq (1), and we can obtain:

$${\left(y-y_{0}\right)}^{2}=b^{2}$$ (23)

Substituting it into Eq (22), the following can be obtained:

$$\Delta d=|\sqrt{{\left(x_{i}-x_{0}\right)}^{2}+{\left(y_{i}-y_{0}\right)}^{2}}-b|$$ (24)

When *x*_*i*_≠*x*_0_, the linear equation of OP’ is:

$$y-y_{0}=\frac{y_{i}-y_{0}}{x_{i}-x_{0}}(x-x_{0})$$ (25)

Substituting it into Eqs (1) and (22), the following can be obtained:

$$\Delta d=\left|\sqrt{{\left(x_{i}-x_{0}\right)}^{2}+{\left(y_{i}-y_{0}\right)}^{2}}-\sqrt{\frac{{\left(x_{i}-x_{0}\right)}^{2}+{\left(y_{i}-y_{0}\right)}^{2}}{{\left(x_{i}-x_{0}\right)}^{2}}{\left(x-x_{0}\right)}^{2}}\right|$$ (26)

where:

$${\left(x-x_{0}\right)}^{2}=\frac{a^{2}b^{2}}{a^{2}+b^{2}{\left(\frac{y_{i}-y_{0}}{x_{i}-x_{0}}\right)}^{2}}$$ (27)

According to the above method, the geometric distance between the identified elliptic domain and the identified AIS data can be calculated [36], which can be regarded as the identification error. The least square method is usually used to solve the problem. The general least square method treats each piece of data as equally important, when the importance of each piece of data may be different. This paper considers that the importance of data of different targets is different. Therefore, when it is necessary to consider the difference in the importance of data, the more reasonable meethod is to use the weighted method. According to the weighted least squares (WLS) identification principle, the identification problem of the ship domain model comes down to the minimum sum of the weighted squares of geometric distances (identification errors) between obtained boundary data points and identified AIS data points. The corresponding minimization objective function is as follows:

$$min\sum_{i=0}^{n}{\left(\Delta d*{CRI}_{i}\right)}^{2}$$ (28)

Eqs (24) and (26) are substituted into Eq (28) to obtain the final weighted objective function as follows:

$$min\sum_{i=0}^{n}\{\begin{array}{l} {\left(|\sqrt{{\left(x_{i}-x_{0}\right)}^{2}+{\left(y_{i}-y_{0}\right)}^{2}}-\sqrt{\frac{{\left(x_{i}-x_{0}\right)}^{2}+{\left(y_{i}-y_{0}\right)}^{2}}{{\left(x_{i}-x_{0}\right)}^{2}}*\frac{a^{2}b^{2}}{a^{2}+b^{2}{\left(\frac{y_{i}-y_{0}}{x_{i}-x_{0}}\right)}^{2}}}|*{CRI}_{i}\right)}^{2}\;x_{i}\neq x_{0}\\ {\left(|\sqrt{{\left(x_{i}-x_{0}\right)}^{2}+{\left(y_{i}-y_{0}\right)}^{2}}-b|*{CRI}_{i}\right)}^{2}\;x_{i}=x_{0} \end{array}$$ (29)

where *CRI*_*i*_ is the ship collision risk corresponding to AIS data points, which is used as the weight of identification error and obtained by the PSO-LSSVM method.

By substituting AIS data into the ship domain model equation and iterating parameters, the objective function in Eq (29) is minimized, and the corresponding ship domain model parameters can be identified.

## 5. Experiments

### 5.1. Experimental instructions

To verify the feasibility of the research method, the dynamic changes in the ship domain during ship navigation are analysed, and the differences in the evolution characteristics of the ship domain in different waters are compared. Therefore, navigation process experiments are carried out in ship routing and other waters. The Yangtze Estuary area, which requires a long voyage in and out of the port, was chosen to allow a sufficiently long time to observe changes. Busy Yangtze River estuary waters can obtain more data to meet the update frequency requirements. In the selection of ship type, the mainstream ship size and ordinary cargo ship type are selected. To better reflect the general situation, dangerous ships with special characteristics should be avoided.

**Fig 6:**
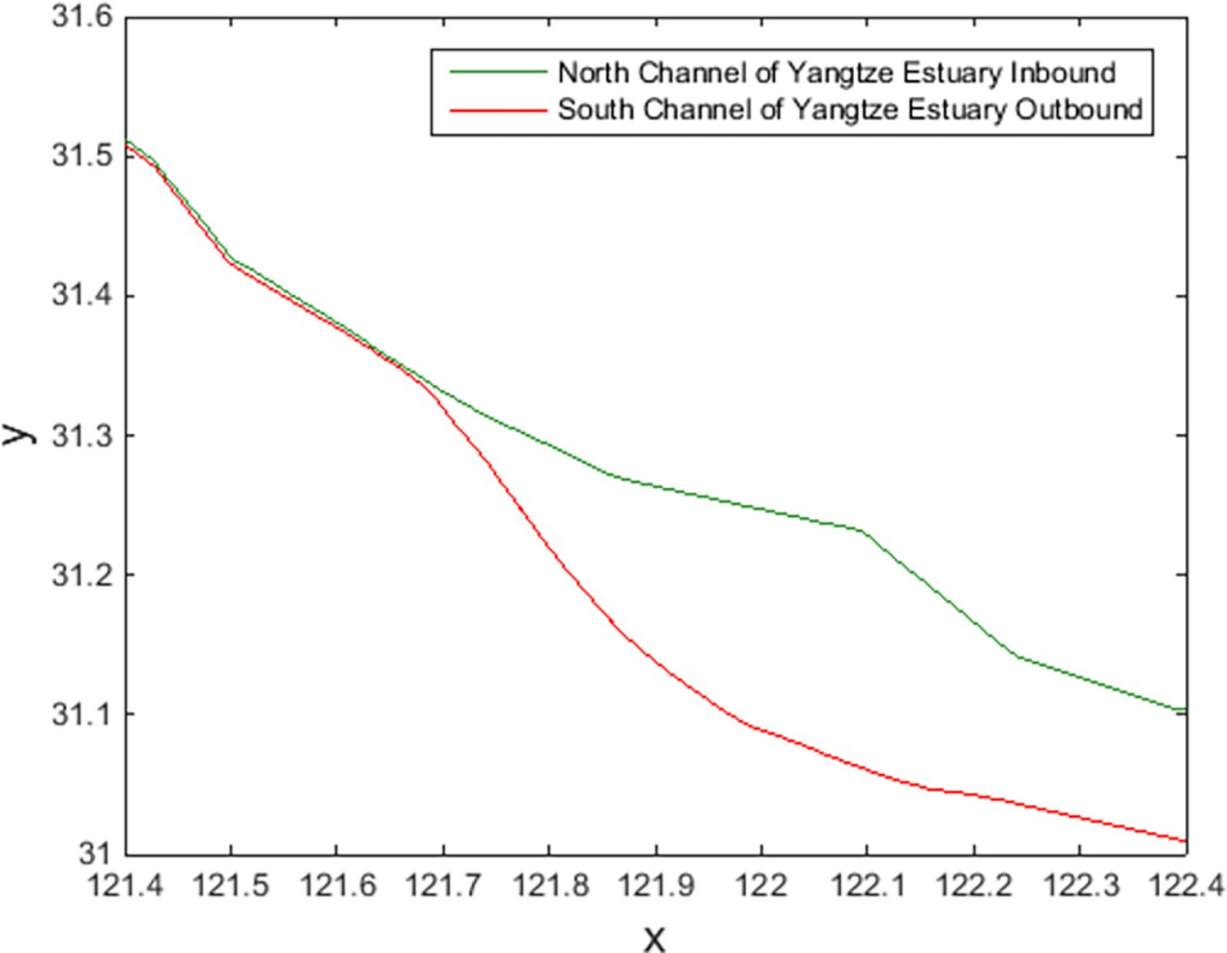
AIS track of ships entering and leaving ports.

In experiments, AIS data are accessed, decoded, and preprocessed; abnormal data are deleted; data within the research scope are screened, and the dynamic overlay is calculated to generate the encounter situation. Before using the PSO-LSSVM method, the data need to be normalized. Then, identification is carried out according to the method presented in this paper. Through the test, it is found that selecting approximately 1 hour of data accumulation can meet the requirements of data volume and data distribution, and approximately 10 minutes of dynamic update can observe significant changes, which basically meets the requirements of dynamics. Two ordinary cargo ships of the same size are selected for the experiment. Periods close to the same day are selected to eliminate weather and other disturbances as much as possible and enhance comparability. For example, a general cargo ship (with a length of 190 m and a width of 32 m) exits the port through the South Channel of the Yangtze River Estuary and enters the port through the north deep-water channel. The AIS track is shown in Fig 6. The AIS data approximately 1 hour before the start of identification were used to train and test the PSO-LSSVM. After the training met the requirements, it was used to obtain the CRI of the target ship during the online identification. To compare the characteristics of the methods, the WLS method in this paper is used to identify the ship domain parameters online, and it is compared with the least square (LS) method. Sixty minutes of AIS data accumulation are adopted, updated, and iterated every ten minutes, rolling real-time online identification of the ship domain.

### 5.2. PSO-LSSVM training and testing for CRI

**Fig 7:**
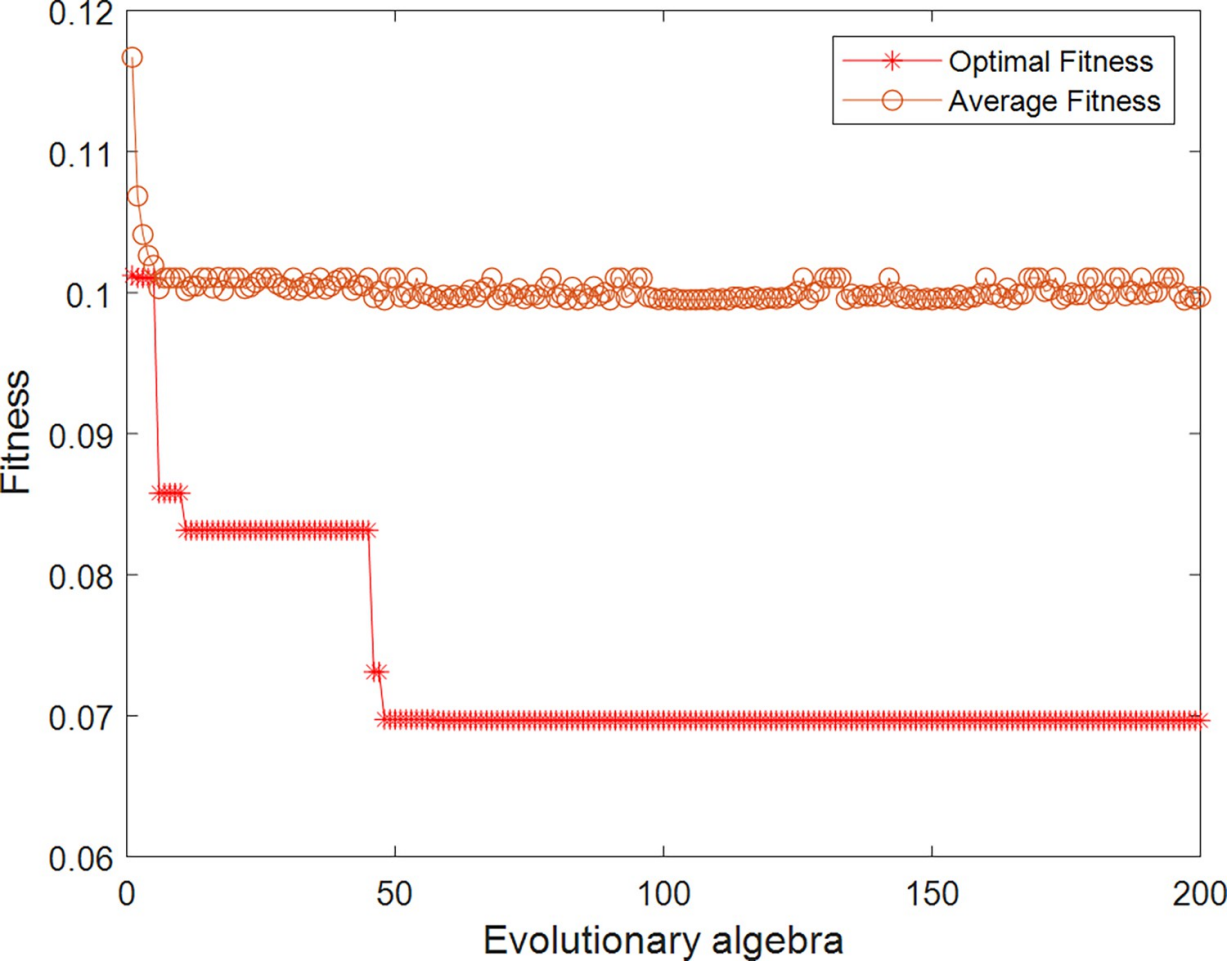
Fitness curve during training.

**Fig 8:**
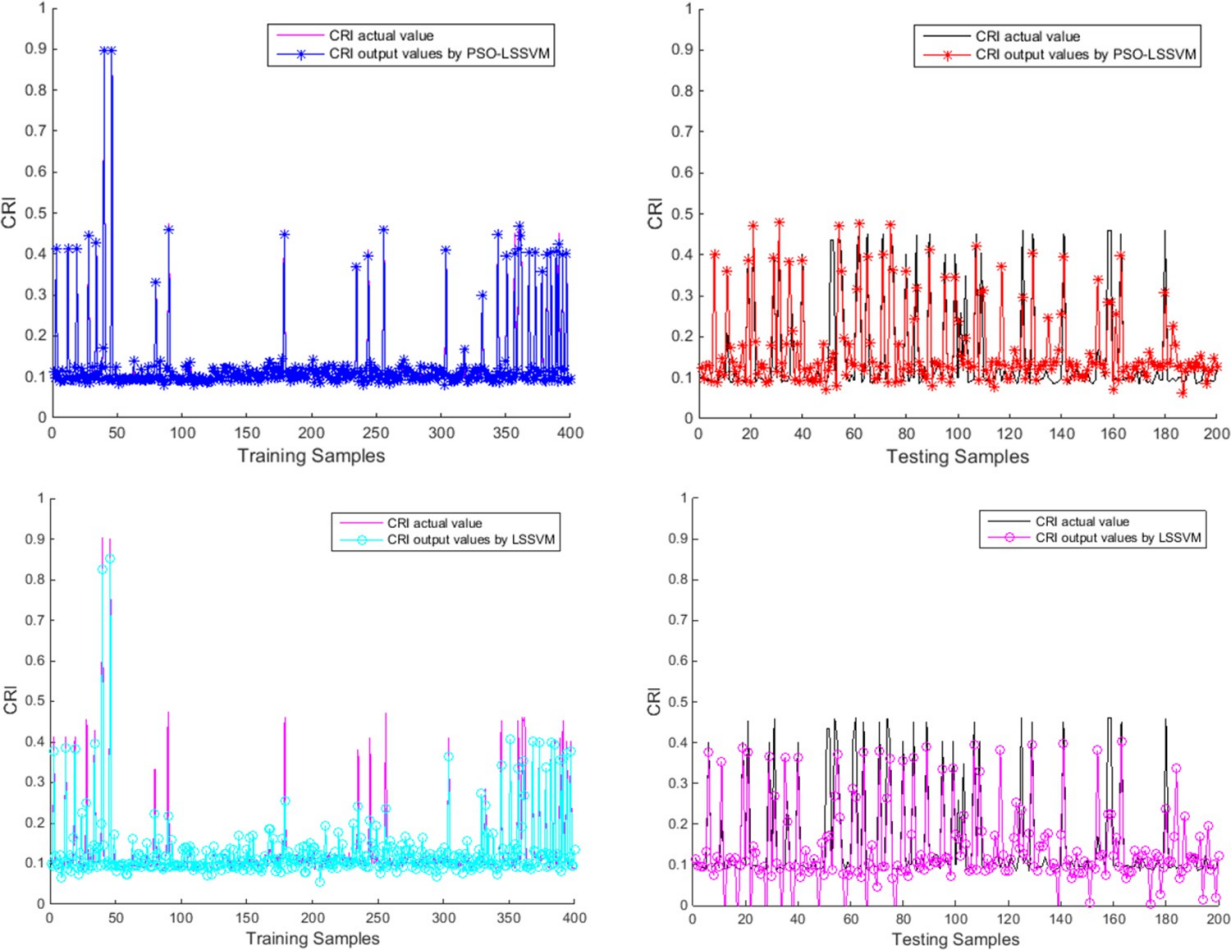
Results of CRI by PSO-LSSVM and comparison of LSSVM results. (a) Training results by PSO-LSSVM (b) Testing results by PSO-LSSVM (c) Training results by LSSVM (d) Testing results by LSSVM.

The previous AIS data of the test area are used to obtain the CRI training set by fuzzy mathematics, and then these data needs to be normalized. After PSO-LSSVM training and testing, the CRI model based on PSO-LSSVM is established to obtain the CRI parameters of the surrounding ships required for ship domain identification. The PSO parameters are set as follows: the learning factors *c*_1_ and *c*_2_ are 1.5 and 1.7 respectively, the momentum coefficient *ω* is 1, the maximum number of evolution is 200, and the size of the particle population is 20. The parameters that need to be optimized include the penalty factor *γ* and the kernel parameter *σ*. After PSO optimization, the optimized parameter *γ* is 100 and *σ*^2^ is 0.1062.Some training samples are shown in Table 2. The fitness in training is shown in Fig 7. The training and testing results of the PSO-LSSVM and a comparison of the LSSVM results are shown in Fig 8.

**Table 2:** Some training samples and results for the CRI-based PSO-LSSVM method.

No	DCPA	TCPA	D	Q	K	CRI (Fuzzy)	CRI (PSO-LSSVM)	Relative Error (PSO-LSSVM)
1	0.62	10.27	3.14	300	0.60	0.0961	0.0965	-0.46%
2	5.45	53.70	13.17	306	0.72	0.0965	0.0966	-0.13%
3	4.83	48.76	12.96	306	0.62	0.0980	0.0979	0.15%
4	0.85	7.24	2.38	131	0.82	0.0859	0.0852	0.77%
5	1.42	12.62	4.11	132	0.80	0.0861	0.0864	-0.33%
6	7.04	25.89	7.61	313	0.86	0.0973	0.0975	-0.23%
7	6.96	26.99	7.61	312	0.85	0.0973	0.0975	-0.25%
8	1.45	29.37	6.61	314	0.34	0.1069	0.1063	0.56%
9	4.95	49.94	11.70	293	0.03	0.1166	0.1171	-0.43%
10	3.68	47.91	10.62	299	0.01	0.1242	0.1248	-0.54%
11	7.05	44.40	14.20	303	0.44	0.1002	0.1006	-0.35%
12	4.38	28.32	7.79	335	0.37	0.1137	0.1140	-0.22%
13	7.16	26.33	7.61	311	0.88	0.0965	0.0961	0.38%
14	6.97	35.33	7.51	310	0.87	0.0963	0.0963	0.04%
15	6.98	35.06	7.51	310	0.88	0.0961	0.0966	-0.55%
16	1.47	24.45	5.44	315	0.36	0.1068	0.1064	0.40%
17	2.31	32.91	6.93	339	0.01	0.1387	0.1387	0.00%
18	9.88	32.57	14.50	351	0.68	0.1129	0.1131	-0.17%
19	3.16	53.30	14.98	353	0.58	0.1148	0.1148	0.06%
20	3.11	23.54	4.75	319	0.66	0.1025	0.1029	-0.33%

In Fig 7, the fitness of PSO decreases gradually during the training process, indicating that the error converges and the training is effective. Fig 8 reflects the comparison between the CRI of output and the CRI of fuzzy calculation. The training results in Fig 8(A) are very close to the original data, and the testing results in Fig 8(B) are also close, so the CRI model established by PSO-LSSVM can be used for subsequent identification. Through the comparison between Fig 8(A) and 8(C), and between Fig 8(B) and 8(D), it can be seen that the PSO-LSSVM has better results than the LSSVM in training and testing.

### 5.3. Online identification outbound process in the south channel of the Yangtze river estuary

According to the AIS data of the ship’s outbound voyage in the South Channel, real-time online rolling identification of ship domain parameters is performed; the dynamic change curves of the ship domain are drawn, and the results are analyzed as follows:**(T6)**

**Fig 9:**
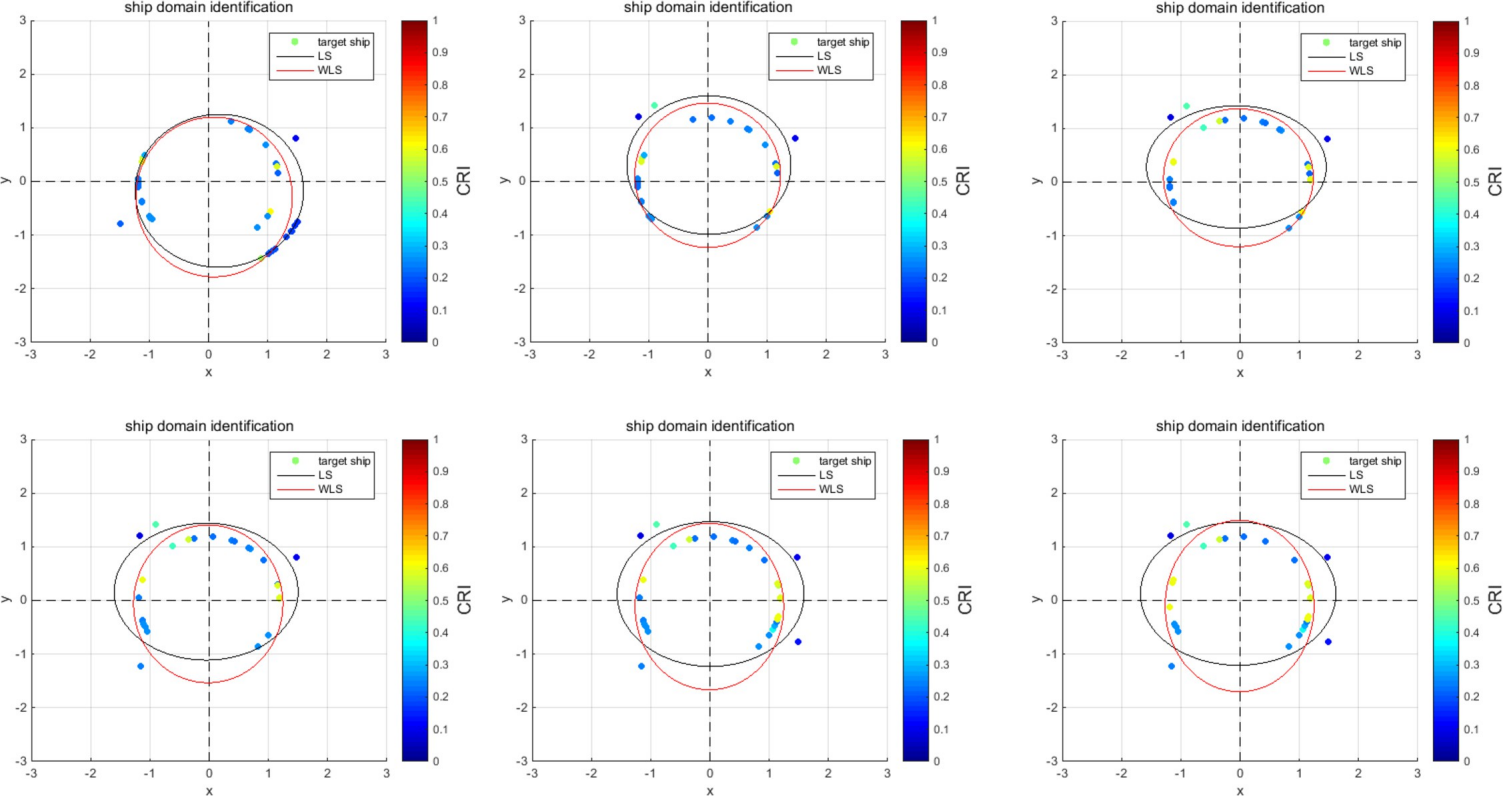
Online rolling identification results of the ship domain. **(T1)** Identification results at Time T1, correspondingly, $\bar{CRI}=0.28$; **(T2)** Identification results at time T2, correspondingly, $\bar{CRI}=0.30$; **(T3)** Identification results at time T3, correspondingly, $\bar{CRI}=0.35$; **(T4)** Identification results at time T4, correspondingly, $\bar{CRI}=0.32$; **(T5)** Identification results at Time T5, correspondingly, $\bar{CRI}=0.35$; **(T6)** Identification results at Time T6, correspondingly, $\bar{CRI}=0.38$.

**Fig 10:**
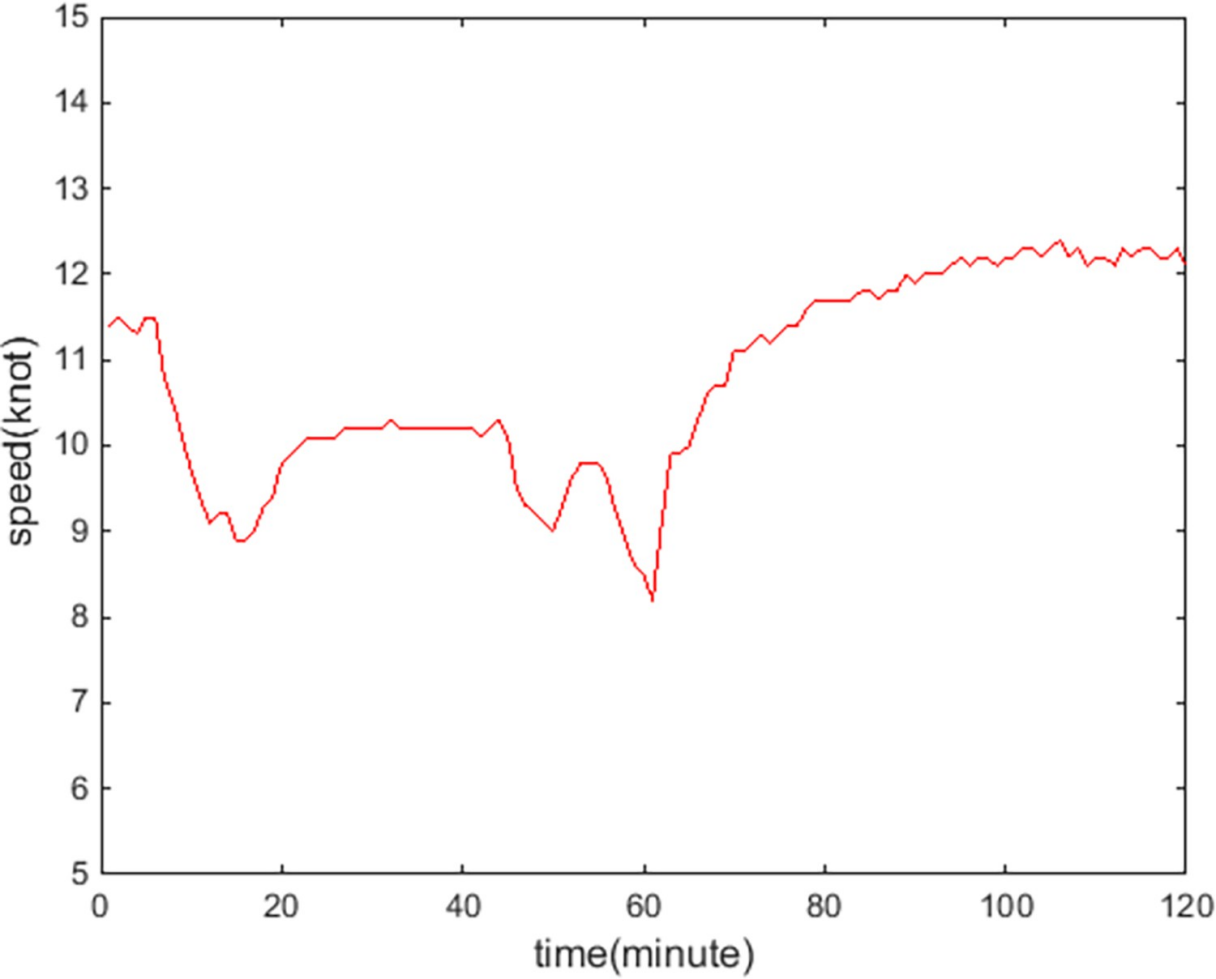
Speed dynamic curve of the own ship.

**Fig 11:**
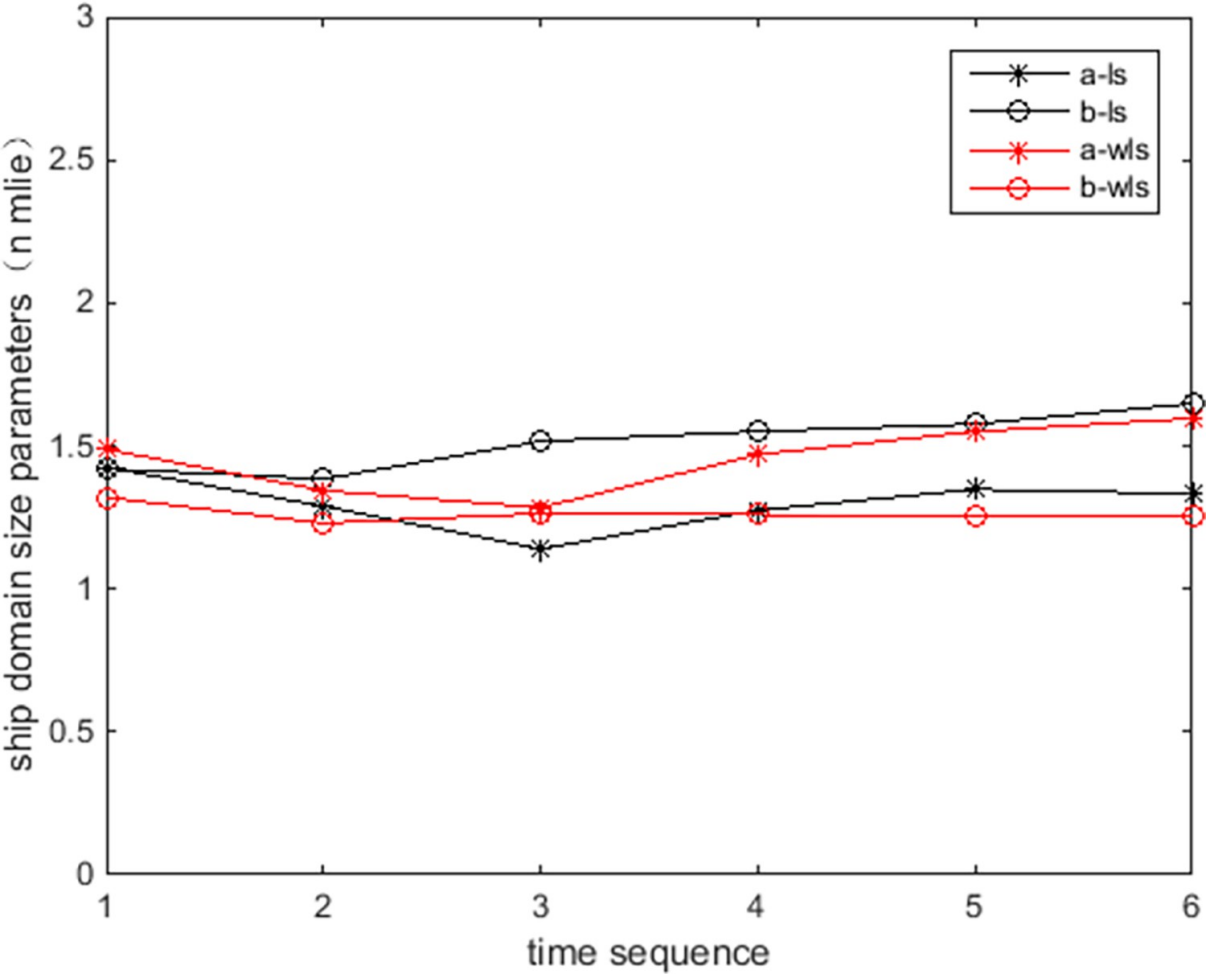
Long and short axes of ship domains by identification.

**Fig 12:**
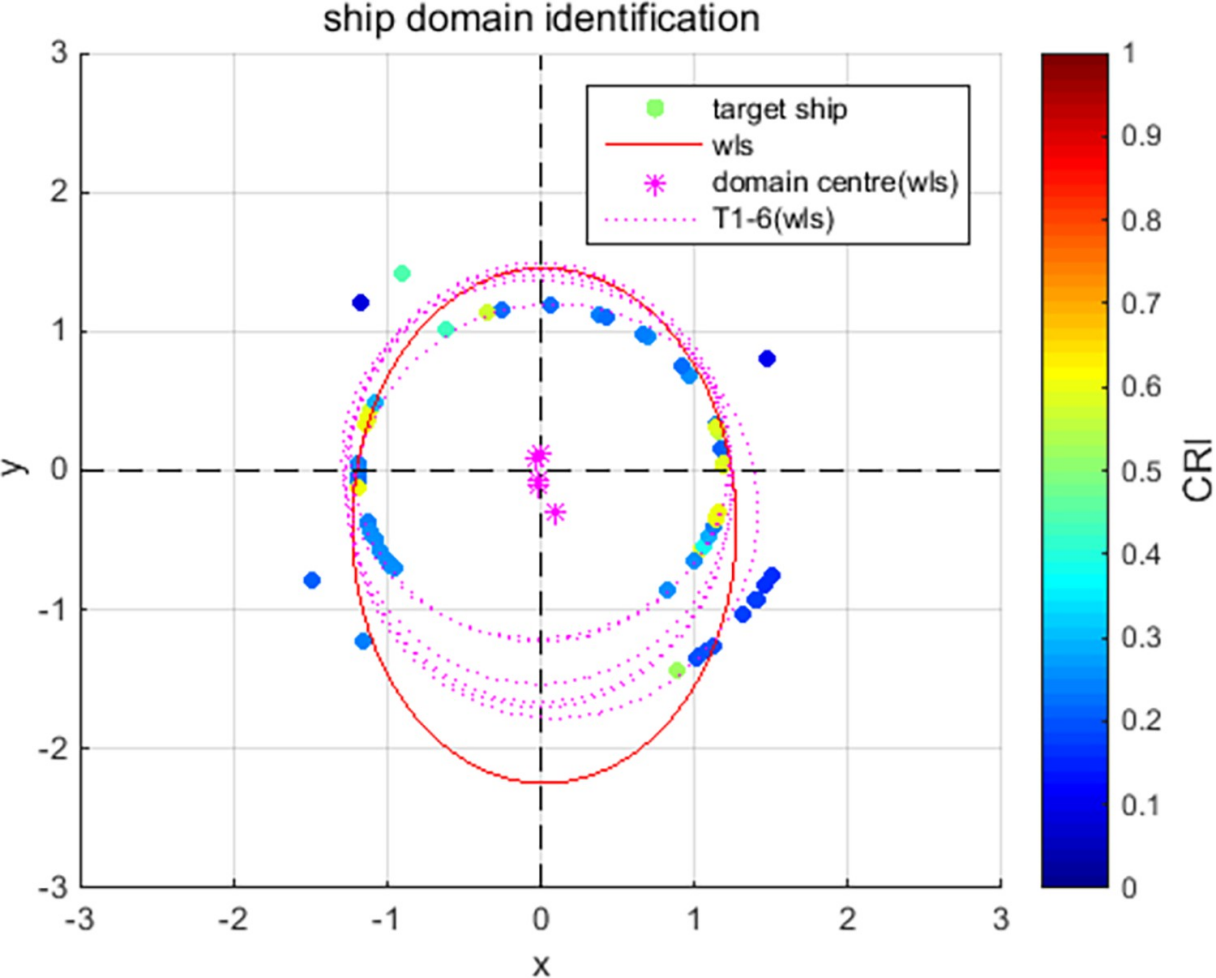
Overlying ship domains by identification.

The results of the outbound process are analyzed as follows:

1. Fig 9(T1) to 9(T6) show the online rolling identification results of the ship domain for one hour when leaving the port from the South Channel and reflect the dynamic evolution process of the ship domain boundary. The South Channel is a water area where the ship’s routing is not implemented, and the boundary process of the ship domain changes obviously during navigation.
2. From Fig 9, there is a significant difference between the results identified by the WLS and LS methods in this paper. The ship domain identified by WLS changed from nearly round to more elongated ellipses. The result of LS identification is a flatter ellipse. The WLS method is less affected by the low-risk target in the transverse position. In contrast, the LS method is obviously affected, and the domain shape is pulled flat, which is inconsistent with the general understanding that the longitudinal length of the ship domain is greater than or equal to the transverse length. In comparison, the WLS method is more suitable for online identification, with an obvious filtering effect of low-risk targets and more accurate identification results.
3. In Fig 9, $\bar{CRI}$ is the average collision risk of the identification targets, which can centrally reflect the reliability of the identification results. The results were more reliable with a higher $\bar{CRI}$. At the same time, $\bar{CRI}$ can also reflect the space that can be compressed in the ship domain. The lower the average value is, the less dangerous targets appear. At present, there is more compression space in the ship domain.
4. Compared with the classic Fujii model, the ship domain presented in this paper is dynamic and correlated with CRI. According to the Fujii model, in narrow waterways, the length of the domain is six times that of the own ship, and the corresponding long axis of the domain is 0.31 nautical miles. In open waters, the length of the domain is eight times that of the own ship, and the corresponding long axis of the domain is 0.41 nautical miles. All of these are obviously smaller than the results in this paper. The Fujii model can be considered as a ship domain with CRI of 1, and then the long-axis results of the two models will sometimes be relatively close. For example, in Fig 9(T1), the long axis of the ship domain is approximately 1.5 nautical miles, and the corresponding $\bar{CRI}$ is 0.28. When CRI is 1, the long axis of the domain is adjusted to 0.42 nautical miles after conversion, which is close to the Fujii model in open water. However, the ship domain model identified in this paper is still larger than the Fujii model, because the pilot will actually maintain a larger safety area when conditions permit, to ensure navigation safety.
5. According to Fig 10, the ship’s speed changes roughly in a pattern of deceleration first and then acceleration. Corresponding to Fig 11, the long axis of the ship domain identified by the WLS method also roughly shows a process of shortening first and then lengthening, while the short axis has no obvious change. This shows that the long axis of the ship domain will become longer with increasing ship speed and will become shorter with decreasing ship speed. The experiment shows that the driver hoped to maintain a larger safety distance with the longitudinal position when the ship speed increased, but the safety distance in the lateral position did not change significantly.
6. In Fig 12, the ship domain overlying diagram shows that the ship domain identified by the WLS method in the dynamic process is roughly within the range of the ship domain identified using the total data accumulation, and its distribution fluctuates. The centers of the ship domain are roughly distributed in the center of the ship, slightly to the right of the rear; in particular, with the increase in ship speed, there is a slightly backward trend.

### 5.4. Online identification inbound process in the north channel of the Yangtze river estuary

According to the AIS data of the ship’s inbound navigation in the North Channel, real-time online rolling identification of ship domain parameters is performed; the dynamic change curves of the ship domain are drawn, and the results are analyzed as follows:**(T6)**

**Fig 13:**
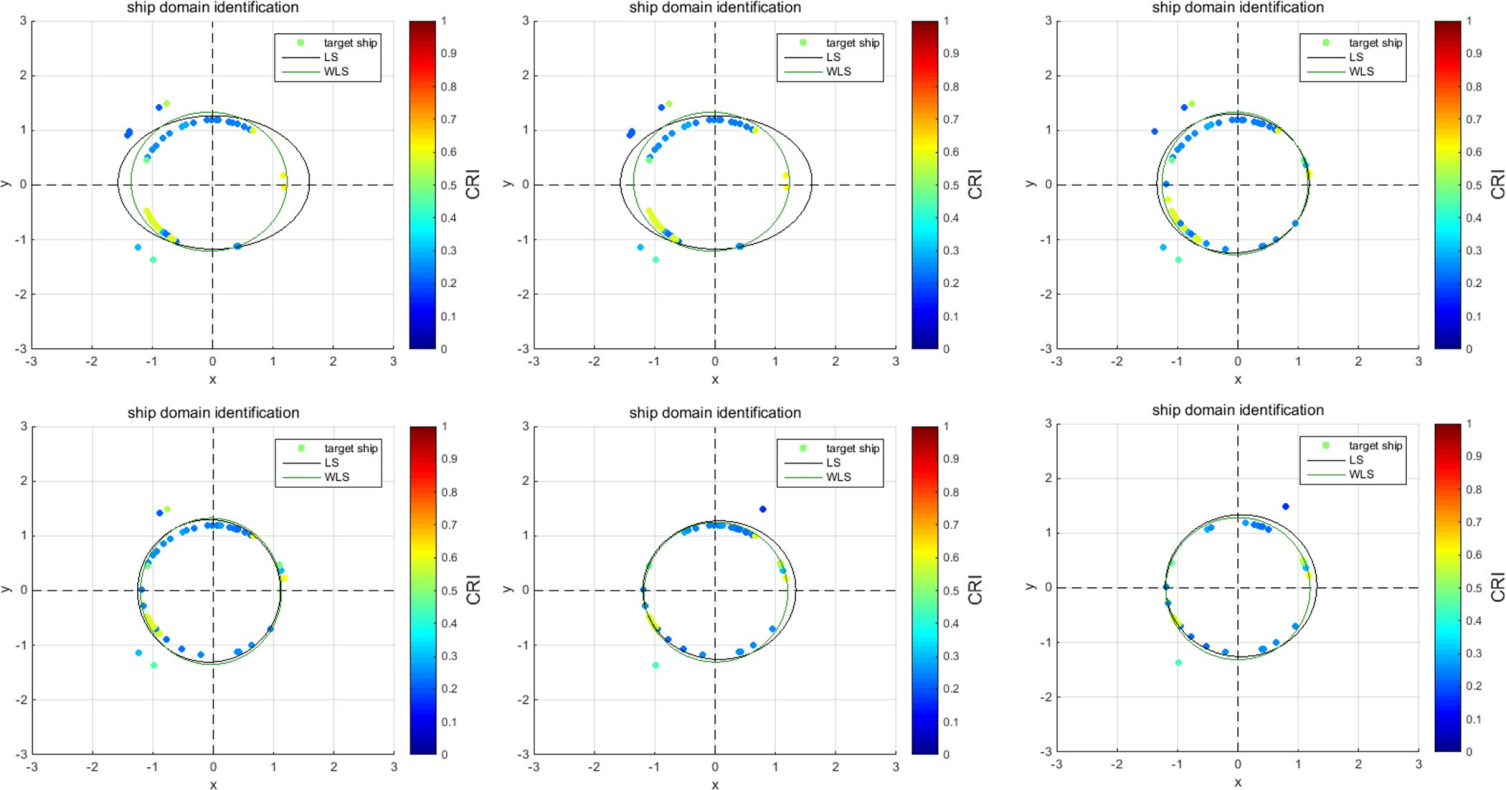
Online rolling identification results of the ship domain. **(T1)** Identification results at Time T1, correspondingly, $\bar{CRI}=0.38$; **(T2)** Identification results at time T2, correspondingly, $\bar{CRI}=0.37$; **(T3)** Identification results at time T3, correspondingly, $\bar{CRI}=0.38$; **(T4)** Identification results at time T4, correspondingly, $\bar{CRI}=0.38$; **(T5)** Identification results at Time T5, correspondingly, $\bar{CRI}=0.39$; **(T6)** Identification results at Time T6, correspondingly, $\bar{CRI}=0.36$.

**Fig 14:**
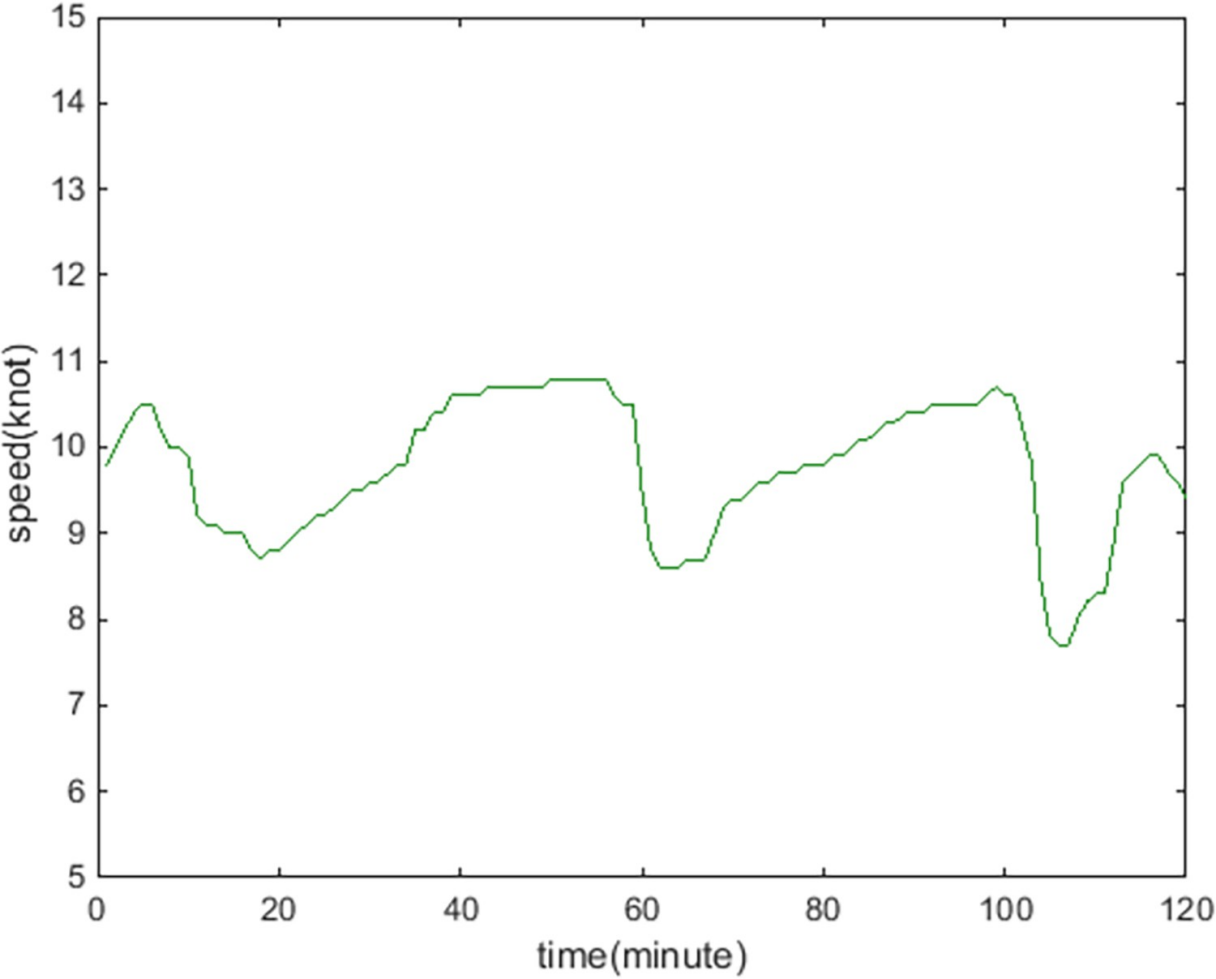
Speed dynamic curve of the own ship.

**Fig 15:**
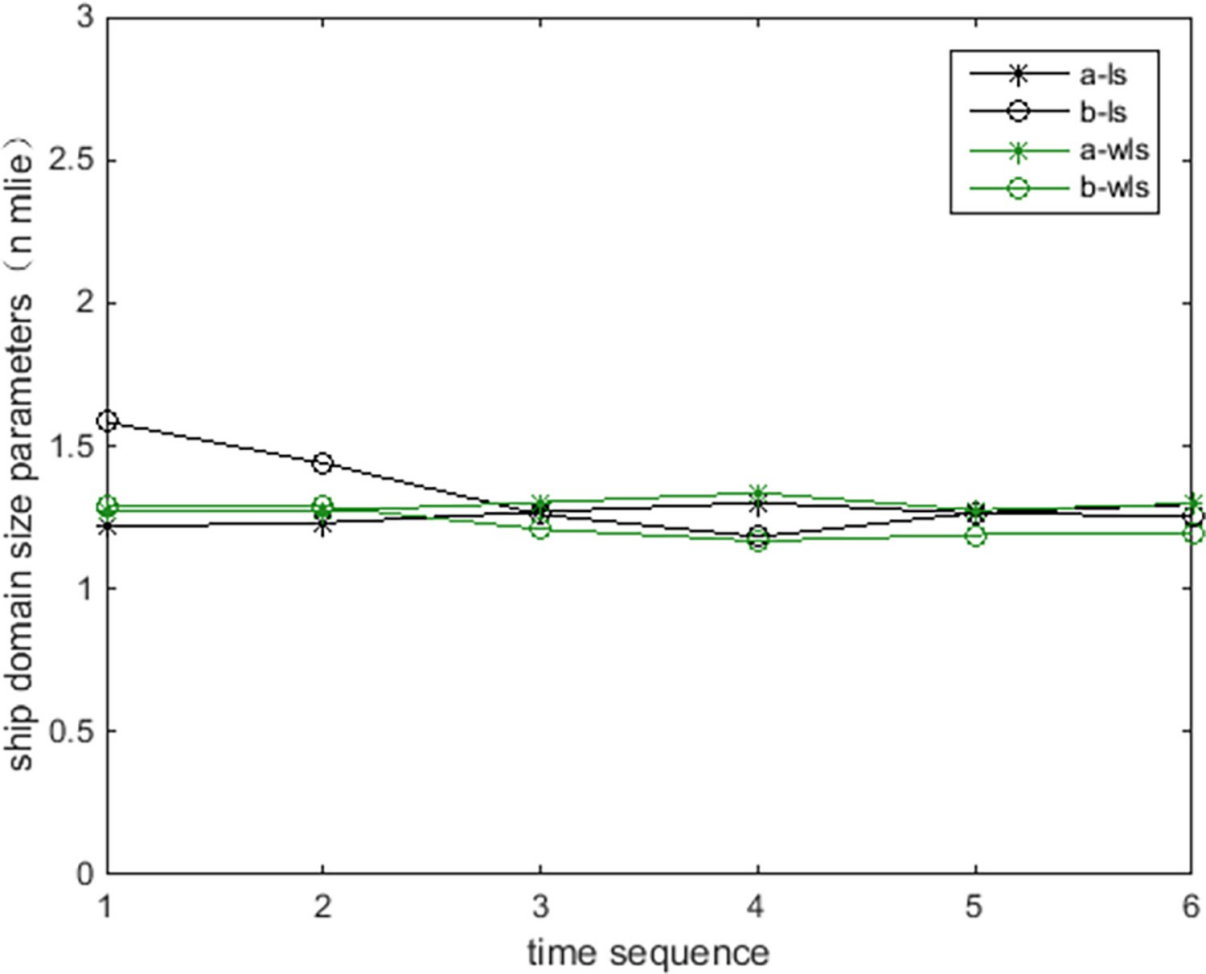
Long and short axes of ship domains by identification.

**Fig 16:**
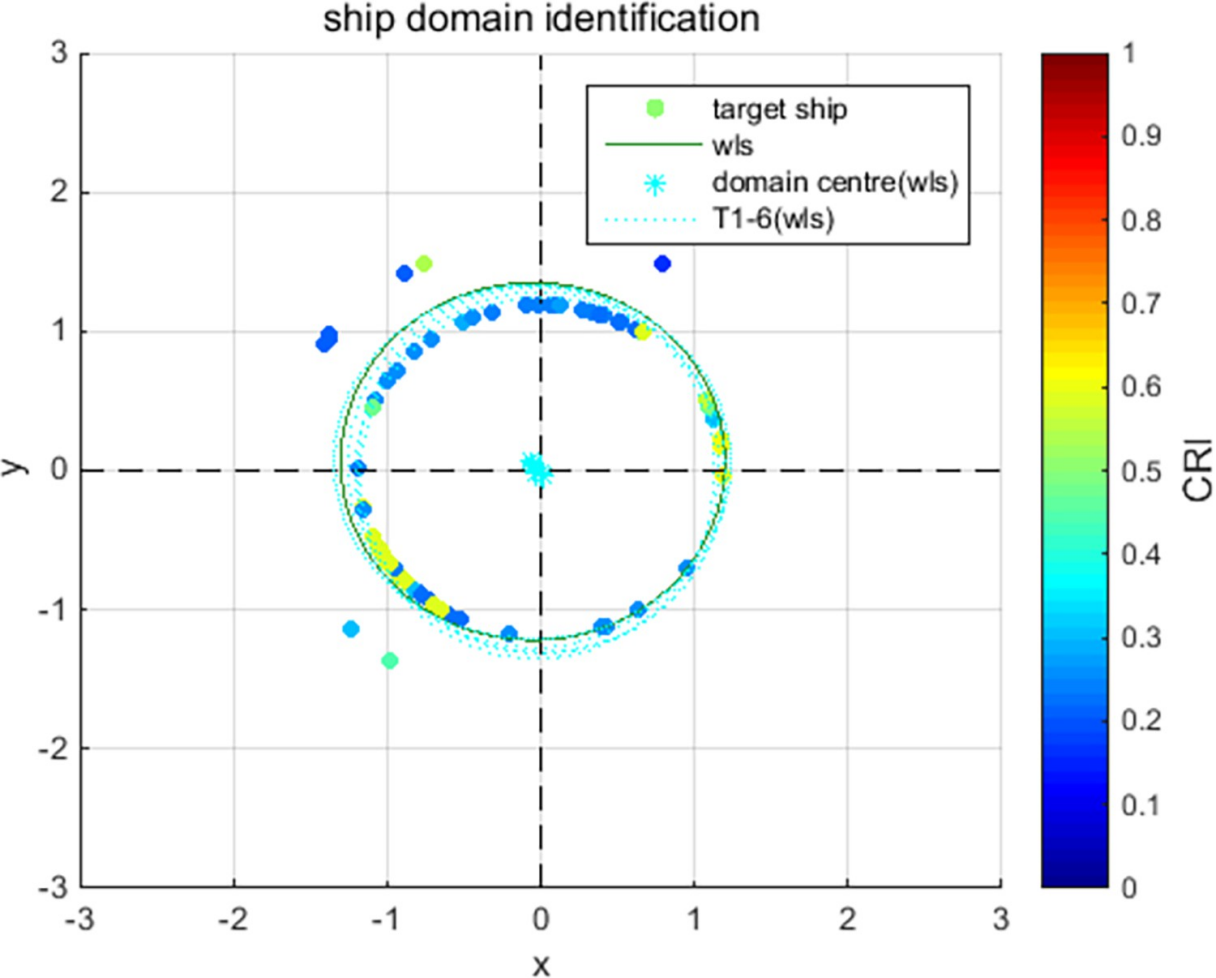
Overlying ship domains by identification.

The results of the inbound process are analyzed as follows:

1. Fig 13(T1) to 13(T6) show the online rolling identification results of the ship domain for one hour when entering the port from the North Channel and reflect the dynamic evolution process of the ship domain boundary. The North Channel is a ship’s routing water area, and the boundary process of the ship domain changes obviously during navigation. The ship navigation efficiency is higher; the ship encounter situation is relatively simpler, and the boundary fluctuation in the ship domain is relatively small and more stable.
2. Fig 13 shows that the identification results of WLS are closer than those of LS, and the domain boundary of WLS identification is smaller. For ships sailing in the North Channel, the inbound time interval is approximately 6 minutes, the speed range is 10–15 knots, and the corresponding inbound distance interval is 1–1.5 nautical miles. The WLS-identified long axis of the ship domain is approximately 1.2 nautical miles, which is roughly consistent with the law of vessel traffic flow.
3. Comparing Fig 13 with Fig 9, $\bar{CRI}$ in the North Channel is larger than in the South Channel, indicating that ships in the North Channel are more closely spaced and that the corresponding identification ship domain is smaller.
4. Fig 14 shows that the ship’s speed fluctuates. In Fig 15, the long axis of the ship domain identified by WLS fluctuates slightly, while the short axis is basically unchanged. The boundary change of the ship domain is not obvious, and it is more influenced by navigation regulations of ship routing.
5. From Fig 16, the ship domain overlying diagram shows that the ship domain identified by the WLS method in the dynamic process is roughly within the range of the ship domain identified using the total data accumulation; the distribution is stable, and the fluctuation is small. The centers of the ship domain are basically distributed in the center of the ship.

## 6. Summary of experiments

Based on the online identification experiment and result analysis of navigation in the South Channel and North Channel of the Yangtze Estuary, it can be summarized as follows:

1. In a water area where the ship’s routing is not implemented, the long axis of the ship domain changes obviously with ship speed, while the short axis is basically unaffected. This shows that with increasing ship speed, the pilot tends to maintain a greater safety distance, and therefore, the boundary of the ship domain is greater. In comparison, in ship-routing waters, the ship domain is more affected by the regulations of navigation, and the domain boundary is more stable. In other words, the fluctuation characteristics of the ship domain are obviously influenced by the traffic environment of sailing waters.
2. Through the overlying diagram of the ship domain, it is found that the ship domain has been changing dynamically in the actual navigation process, with the change in the ship’s motion parameters, the surrounding environment of the navigation waters, and the encounter situation of the own ship and other ships, showing certain correlation, volatility, and randomness.
3. The PSO-LSSVM method is used to obtain CRI, and the collision risk weighting method proposed in this paper can effectively filter nonhazardous targets and improve the impact of dangerous targets on the results, which is consistent with the concept of the ship domain, and its identified ship domain is more consistent with the actual situation of navigation.
4. Through dynamic online rolling identification in the experiment, ship domain parameters can be quickly calculated; the dynamic evolution process of the ship domain in the navigation process can be better presented, and its dynamic fluctuation characteristics and change rules can be found.

The online identification method in this paper introduces risk weighting, which will make the ship domain obtained contain some subjectivity, because the collision risk concept has some subjectivity. In addition, the experiment was carried out in the specific water area, which has a certain representativeness. The corresponding results reflect the evolution characteristics of the ship domain during ship navigation. However, the ship domain is affected by many factors, such as navigation rules, the natural environment and traffic conditions, etc. Different waters will differ in these respects. Therefore, the characteristics summarized in experiments can be used as a reference for other similar waters, but the characteristics of the ship domain in specific waters need to be analyzed through specific experiments.

## 7. Conclusion

Based on real-time AIS data and through the short data accumulated, an online identification method can dynamically generate the ship model by calculating ship-encounter parameters and acquiring the risk of collision as identification data sources to ensure identification accuracy and real-time requirements. By selecting representative waters, the characteristics of the ship domain in ship-routing waters and unimplemented ship-routing waters are analyzed experimentally. It is found that the ship domain changes dynamically in the sailing process, and to some extent with the waters environment and ship motion parameters, which can provide a reference for the study of similar waters.

The method in this paper enables AIS data to be applied to navigation safety decisions in a more real-time manner, explores the dynamic evolution law of the ship domain, and promotes the better application of relevant theories in the ship domain to intelligent navigation. In this paper, dynamic, real-time and procedural research methods are proposed to lay a foundation for research on dynamic characteristics in the ship domain. In the future, it will be possible to study ship domains in complex scenes, such as analyzing the change characteristics of ship domains with ship navigation environments by considering weather conditions, visibility, wind direction, sea state, water depth, and tide, etc.

## Supporting information

S1 File PSO-LSSVM training and testing data. The training and testing data in the file can be used to train the PSO-LSSVM to obtain CRI and plot the results as shown in Figs 7 and 8.
(XLSX)

S2 File Online identification outbound or inbound process data. The own ship’s trajectory can be plotted using the position data, as shown in Fig 6. The outbound data in Excel can be used for rolling online recognition, and the results can be obtained as shown in Figs 9 to 12. Meanwhile, the inbound data in Excel can be used for rolling online recognition, and the results can be obtained as shown in Figs 13 to 16.
(XLSX)
